# Impact of Combined Exposure to Copper Nanoparticles, Copper Oxide Nanoparticles, and Pesticides on the Metabolic Activity of *Nitrobacter winogradskyi*

**DOI:** 10.3390/ijms26136391

**Published:** 2025-07-02

**Authors:** Roberto Gajardo, Olga Rubilar, Edgar López-Mena, Gildardo Sanchez-Ante, Paola Fincheira, Miguel Martinez, Mauricio Schoebitz, Ricardo Tighe-Neira, Claudio Inostroza-Blancheteau, Leonardo Bardelhe, Gonzalo Tortella-Fuentes

**Affiliations:** 1 Department of Microbiology, Faculty of Biological Sciences, Universidad de Concepción, Concepción 4070386, Chile; rgajardo@udec.cl (R.G.); mimartin@udec.cl (M.M.); 2 Chemical Engineering Department, Universidad de La Frontera, Temuco 4780000, Chile; olga.rubilar@ufrontera.cl (O.R.); paola.fincheira@ufrontera.cl (P.F.); 3 Centro de Excelencia en Investigación Biotecnológica Aplicada al Medio Ambiente (CIBAMA-BIOREN), Casilla 54-D, Temuco 4780000, Chile; 4 Tecnológico de Monterrey, Campus Guadalajara, Av. Gral Ramón Corona No 2514, Colonia Nuevo México, Zapopan, Jalisco 45121, México; edgarl@tec.mx (E.L.-M.); gildardo.sanchez@tec.mx (G.S.-A.); 5 Department of Soil and Natural Resources, Faculty of Agronomy, Universidad de Concepción, Concepción 4030000, Chile; mschoebitz@udec.cl; 6 Biotechnology Center, Renewable Resources Laboratory, Universidad de Concepción, Concepción 4030000, Chile; 7 Departamento de Ciencias Agropecuarias y Acuícolas, Facultad de Recursos Naturales, Universidad Católica de Temuco, P.O. Box 15-D, Temuco 4780000, Chile; rtighe@uct.cl (R.T.-N.); claudio.inostroza@uct.cl (C.I.-B.); 8 Departamento de Producción Agropecuaria, Facultad de Ciencias Agropecuarias y Medioambiente, Universidad de La Frontera, Av. Francisco Salazar 01145, Casilla 54-D, Temuco 4811230, Chile

**Keywords:** copper nanoparticles, pesticides, combined pollution, nitrobacter, metabolic activity

## Abstract

Copper nanoparticles (CuNPs) are increasingly used in agriculture either alone or in combination with pesticides. Recognizing the potential hazards of CuNPs in soil environments, our study evaluated their effects on the metabolic activity of *Nitrobacter winogradskyi* ATCC 2539, a chemolithoautotrophic bacterium crucial for the nitrification process, which involves the oxidation of nitrite to nitrate in soil ecosystems. This study evaluated the effects of concentration ranges of CuNPs (2.5 to 162.7 mg L^−1^), CuONPs (3.2 to 203.6 mg L^−1^), and various pesticides (iprodione, carbendazim, and 2,4-D) and their derivatives (3,5-dichloroaniline, catechol, and 2,4-dichlorophenol) at concentrations ranging from 0.04 to 2.56 mM. CuSO_4_ was also used as a control for comparative purposes. Our findings indicated that the CuNPs significantly inhibited the metabolic activity of *N. winogradskyi*, resulting in a reduction of up to 95% at concentrations of ≥2.5 mg L^−1^. The CuONPs were less toxic, while the pesticides and their derivatives generally showed lower toxicity. Notably, combinations of CuNPs with pesticides or their derivatives maintained high toxicity levels comparable to those of the CuNPs alone. According to the Loewe additivity model, these effects were largely additive and primarily associated with CuNPs or CuONPs. Protein profiling using matrix-assisted laser desorption/ionization (MALDI) time-of-flight (TOF)/TOF mass spectrometry (MS) revealed that carbendazim induced noticeable changes in protein profiles. These findings underscore the detrimental impacts of CuNPs and CuONPs on the metabolic activity of *N. winogradskyi*, posing a considerable risk to the health of agricultural soils. Overall, this research provides crucial insights into the risks associated with using CuNPs in agriculture, particularly regarding their potential threat to nitrifying microorganisms in soils.

## 1. Introduction

Pests in agriculture have a significant impact on global crop yields, with losses exceeding 20% in major crops [1]. Addressing this issue requires the development of effective and environmentally friendly pesticides, which remains a critical challenge. Recently, copper nanoparticles (CuNPs) have emerged as promising antimicrobial agents and slow-release psychostimulants. They offer enhanced performance in plant disease control compared to traditional copper-based compounds, such as copper sulfate. Nanoparticles (NPs) are innovative technologies with unique chemical and physical properties that differ from those of their bulk counterparts [2]. However, these properties may also impact soil’s health and microbiota, raising environmental concerns [3].

Recent research efforts have concentrated on regulating copper sources to minimize copper accumulation in agricultural soils [4] and developing new copper-based engineered nanoparticles. These advancements aim to enhance efficacy while reducing the copper load in soil. Despite these technological improvements, a tendency persists in applying copper compounds at potentially hazardous concentrations without adequately considering their environmental impacts [5].

Investigating the soil accumulation of CuNPs presents a complex challenge due to intrinsic factors, including size, charge, composition, and shape, as well as external factors, such as pH, organic matter content, soil type, and humidity. These factors collectively influence the behavior of CuNPs and/or copper oxide nanoparticles (CuONPs) in soil, affecting the release rate of ionic copper species [6,7], the total exchangeable Cu^2+^, and the bioavailability of copper to various soil organisms [8].

Furthermore, CuNPs and CuONPs may induce changes in bacterial metabolism, alter the richness of bacterial communities, and impact key soil processes such as ammonification, nitrification, and denitrification [9]. Soil nitrification, a vital component in plant nutrition, is primarily mediated by ammonia-oxidizing bacteria (AOB), nitrite-oxidizing bacteria (NOB), and ammonia-oxidizing archaea (AOA), which control the ammonia-to-nitrate conversion rates in soils [10,11]. *Nitrobacter*, along with other genera such as *Nitrospira*, *Nitrococcus*, and *Nitrotoga*, represents a key group of nitrite-oxidizing bacteria (NOB) involved in the nitrification process. *Nitrobacter* is often used as a model organism due to its well-characterized physiology and sensitivity to environmental stressors, and it is particularly sensitive to chemical agents in soil, including heavy metals, pesticides, and metallic nanoparticles [12,13,14]. However, detailed investigations into the impacts of CuNPs or CuONPs on nitrifying bacteria remain limited [15].

Moreover, there is growing evidence that CuNPs and CuONPs can alter the absorption and mobility of pesticides in soil [16,17]. However, comprehensive studies detailing the simultaneous effects of pesticide exposure and CuNPs on the nitrification process are scarce [18]. This study aimed to evaluate the impact of increasing concentrations of mixtures of copper nanoparticles (CuNPs or CuONPs) with various pesticides (iprodione, carbendazim, and 2,4-D) and their derivatives (3,5-dichloroaniline, catechol, and 2,4-dichlorophenol) on the metabolic activity of *Nitrobacter winogradskyi* ATCC 25391.

## 2. Results

### 2.1. Metabolic Activity

**Figure 1:**
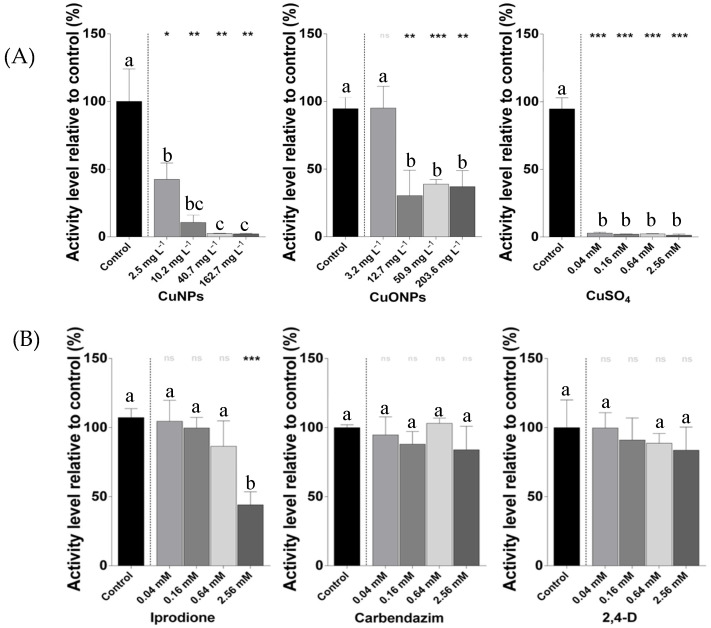
Metabolic activity of *Nitrobacter winogradskyi* after 8 days of exposure to different treatments. (**A**) Copper-based compounds: copper nanoparticles (CuNPs), copper oxide nanoparticles (CuONPs), and copper sulfate (CuSO_4_). (**B**) Agrochemicals: fungicides (iprodione and carbendazim) and herbicide 2,4-D. The bars represent the mean values ± standard deviations (SDs) from the triplicate assays. The different lowercase letters above the bars indicate the statistically significant differences among the concentrations within each treatment group, as determined by one-way ANOVA followed by a Tukey’s post hoc test (*p* < 0.05). The asterisks indicate the statistically significant differences compared to the control, based on paired Student’s *t*-tests (* *p* < 0.05; ** *p* < 0.01; and *** *p* < 0.0001).

The treatments with CuNPs (Figure 1A) showed significant decreases in the metabolic activity of *Nitrobacter winogradskyi* at all tested concentrations, as determined by one-way ANOVA followed by a Tukey’s post hoc test. The CuONPs also caused reductions in metabolic activity, particularly at intermediate and high concentrations, although their effects were less pronounced than those observed with the CuNPs. In contrast, the CuSO_4_ induced the strongest and most consistent inhibition across all concentrations. Regarding agrochemicals (Figure 1B), iprodione exhibited a significant inhibitory effect only at the highest concentration (2.56 mM), whereas no significant differences among the concentrations were detected for carbendazim and 2,4-D.

**Figure 2:**
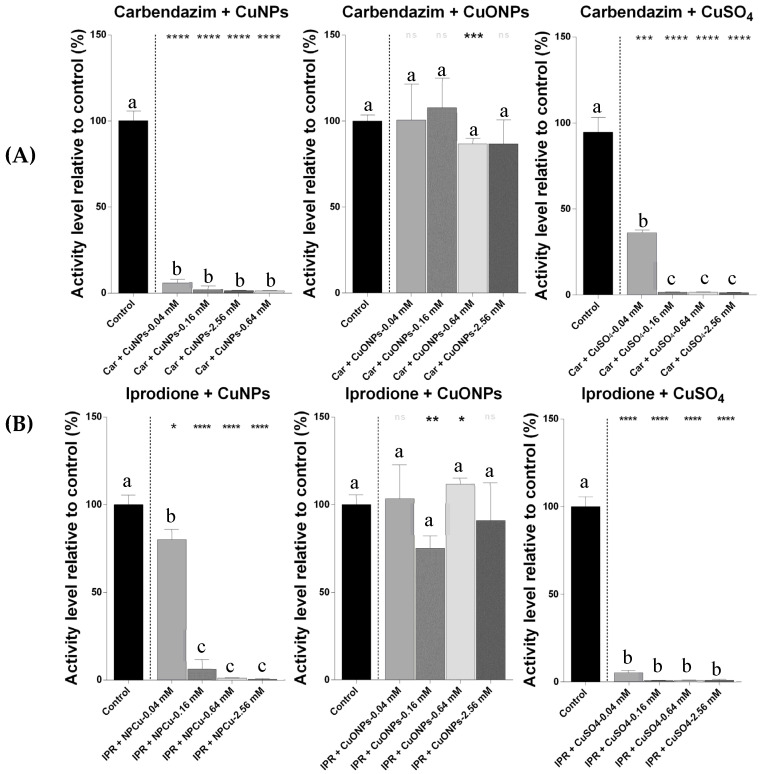
Metabolic activity of *nitrobacter winogradskyi* after 8 days of exposure to the combined treatments with fungicides and copper-based compounds. (**A**) Carbendazim combined with copper nanoparticles (CuNPs), copper oxide nanoparticles (CuONPs), and copper sulfate (CuSO_4_). (**B**) Iprodione combined with copper nanoparticles (CuNPs), copper oxide nanoparticles (CuONPs), and copper sulfate (CuSO_4_). The bars represent the mean values ± SDs from the triplicate assays. The different lowercase letters (a, b, c) indicate the statistically significant differences among the treatments, according to the one-way ANOVA and Tukey’s post hoc test (*p* < 0.05). The asterisks indicate the statistically significant differences compared to the control, based on paired Student’s *t*-tests (* *p* < 0.05; ** *p* < 0.01; *** *p* < 0.001; **** *p* < 0.0001; ns: not significant difference).

Similarly, the combined treatments involving carbendazim or iprodione with the CuNPs or CuSO_4_ resulted in significant reductions in metabolic activity (Figure 2). These treatments were statistically grouped apart from the control and single-compound exposures according to the Tukey’s test, reflecting strong interaction effects. In contrast, combinations with the CuONPs generally produced milder effects and were often not statistically different from the control group or the individual pesticide treatments.

**Figure 3:**
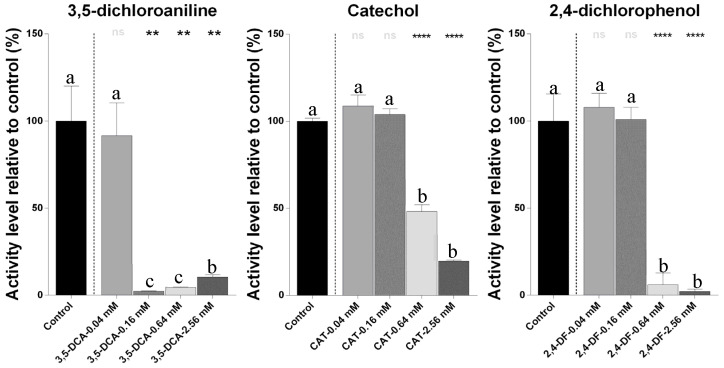
Metabolic activity of *N. winogradskyi* after 8 days of exposure to pesticide derivative compounds (catechol, 3,5-dichloroaniline, and 2,4-dichlorophenol) at different concentrations. The bars represent the means ± SDs. The different lowercase letters (a, b, c) indicate the statistically significant differences among the concentrations for each compound, as determined by one-way ANOVA followed by a Tukey’s post hoc test (*p* < 0.05). The asterisks indicate the statistically significant differences compared to the control, based on paired Student’s *t*-tests (** *p* < 0.01; and **** *p* < 0.0001).

The pesticide derivative 3,5-dichloroaniline showed a significant reduction in metabolic activity, especially at 0.16 mM, while catechol and 2,4-dichlorophenol exhibited significant (*p* < 0.05) inhibitory effects at higher concentrations (Figure 3). According to the Tukey’s test, the highest concentrations of each compound clustered in groups were significantly (*p* < 0.05) different from the controls and lower concentrations, indicating dose-dependent effects.

**Figure 4:**
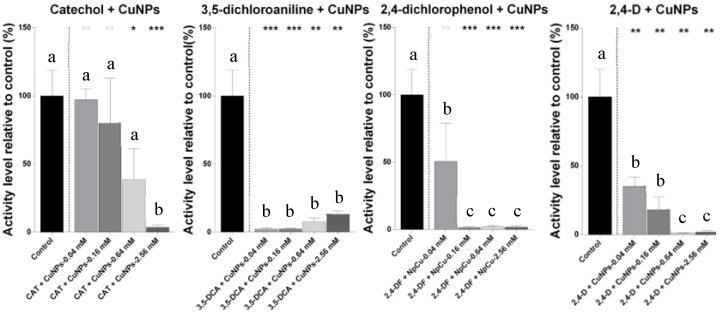
Metabolic activity of *N. winogradskyi* after 8 days of exposure to combinations of the pesticide derivative compounds (catechol, 3,5-dichloroaniline, and 2,4-dichlorophenol), herbicide 2,4-D, and copper nanoparticles (CuNPs) at various concentrations. The bars represent the means ± SDs from the triplicate assays. The different lowercase letters (a, b, c) above the bars denote the statistically significant differences among the treatments, as determined by one-way ANOVA followed by a Tukey’s post hoc test (*p* < 0.05). The asterisks indicate the statistically significant differences compared to the control, based on paired Student’s *t*-tests (* *p* < 0.05; ** *p* < 0.01; and *** *p* < 0.001).

In the case of the combined treatments with pesticide derivatives and CuNPs, significant reductions in metabolic activity were observed for most of the mixtures tested (Figure 4). Treatments such as 2,4-D + CuNPs, catechol + CuNPs, and 3,5-dichloroaniline + CuNPs were assigned to statistically distinct groups compared to the controls and to the respective single-compound treatments, as determined by ANOVA and the Tukey’s post hoc analysis. This highlighted the enhanced toxicity of certain mixtures, even at low concentrations.

### 2.2. IC_50_ Estimation of the Effects of Several Concentrations of Pesticides with Nanoparticles

Table 1 illustrates the relative toxicity of the tested compounds, as determined by their IC_50_ values. The CuONPs were less toxic than the CuNPs, while the CuNPs exhibited similar toxicity levels to the CuSO_4_. Regarding the pesticides, iprodione revealed more toxicity than carbendazim, but carbendazim showed equivalent toxicity to 2,4-D. Among the pesticide derivatives, 3,5-dichloroaniline was more toxic than catechol, whereas 2,4-dichlorophenol was less toxic than catechol. When considering the single treatments, the copper-based compounds displayed more significant toxicity than the pesticide derivatives, but overall, the pesticides were less harmful than the copper-based compounds.

For the mixed treatments, the combinations of carbendazim with the CuNPs and CuSO_4_ were more toxic than those with the CuONPs. A similar pattern was observed for iprodione when mixed with the CuNPs and CuSO_4_, as opposed to its combination with the CuONPs. Interestingly, the treatments of iprodione mixed with the CuONPs were less toxic than the individual treatments of iprodione or the CuONPs. When the CuNPs were mixed with the pesticide derivatives, these combinations resulted in higher toxicity than the single pesticide derivative treatments. Specifically, treatments of catechol mixed with the CuNPs were less toxic than those of 3,5-dichloroaniline mixed with the CuNPs, while combinations of 2,4-dichlorophenol with the CuNPs were more toxic than those of 3,5-dichloroaniline with the CuNPs.

### 2.3. The Loewe Additive Model and Scheirer–Ray–Hare Test

**Figure 5:**
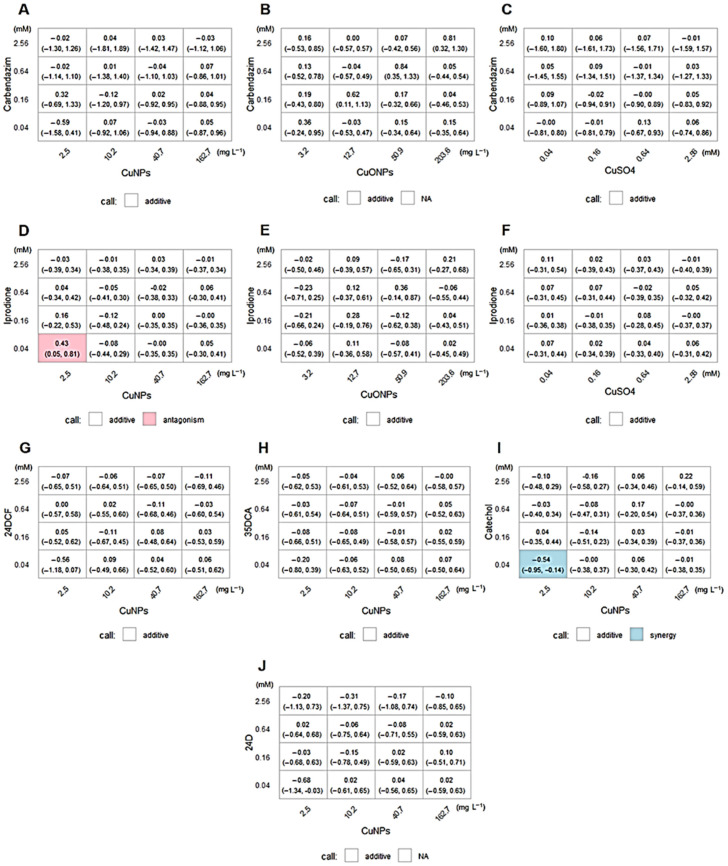
Isobolograms based on the Loewe additivity model for the combined treatments. Panels (**A**–**C**) correspond to the combinations of carbendazim with the CuNPs (**A**), CuONPs (**B**), and CuSO_4_ (**C**). Panels (**D**–**F**) show iprodione with the CuNPs (**D**), CuONPs (**E**), and CuSO_4_ (**F**). Panels (**G**–**J**) represent combinations of the CuNPs with the following pesticide derivatives: 3,5-dichloroaniline (**G**), catechol (**H**), 2,4-dichlorophenol (**I**), and 2,4-D (**J**). Each heatmap illustrates the deviation from additivity, with red indicating antagonistic effects, white indicating additive interactions, and blue indicating synergistic interactions. The concentration ratios and normalized biological responses were plotted to visualize the nature of the compound interactions.

The Loewe additivity model analysis (Figure 5) revealed interaction patterns between the Cu-based compounds and the pesticides or their derivatives. In the carbendazim series (panels A–C), most combinations exhibited additive effects, except for the carbendazim + CuONPs, which showed a tendency toward antagonism at higher concentrations. In the iprodione series (panels D–F), the mixture of iprodione with the CuNPs displayed an antagonistic interaction at the lowest concentration tested (0.04 mM + 2.5 mg L^−1^), while the other combinations were predominantly additive. In the case of the pesticide derivatives (panels G–J), the combination of CuNPs + catechol resulted in a synergistic interaction, suggesting enhanced inhibitory effects. The combinations with 3,5-dichloroaniline, 2,4-dichlorophenol, and 2,4-D were mostly additive. These results supported the interpretation that the copper nanoparticles were the main drivers of metabolic inhibition, with specific interactions depending on the chemical nature of the co-applied compound.

### 2.4. MALDI TOF/TOF MS

**Figure 6:**
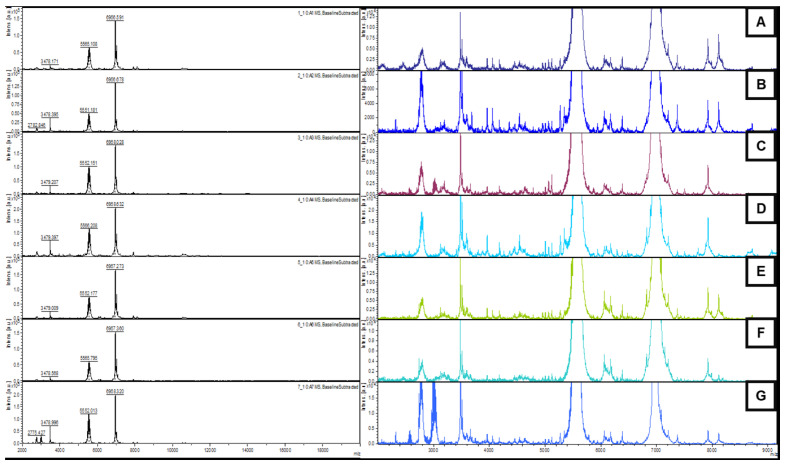
The protein profiles of *N. winogradskyi* obtained by MALDI-TOF MS/MS generated by exposure to the treatments. **Left**: main profiles. **Right**: details of the sub-represented peaks. (**A**) control, (**B**) the CuNPs, (**C**) carbendazim, (**D**) iprodione, (**E**) the CuONPs, (**F**) iprodione + CuNPs, and (**G**) carbendazim + CuNPs.

All samples exhibited three dominant peaks at 3478, 5565, and 6956 *m*/*z*, and this was common to all the treatments (Figure 6, left), indicating the core proteins expressed by *N. winogradskyi*. However, noticeable changes in the presence and intensity of additional peaks were detected across the treatments (Figure 6, right). The CuNPs and carbendazim treatments showed the highest number of additional peaks not present in the control, suggesting stress-induced protein expression. In contrast, the CuONPs and control groups displayed very similar profiles with minimal additional peaks. These differences in protein fingerprinting indicated that the exposure to the CuNPs or carbendazim triggered significant metabolic or structural changes in the bacterium, consistent with the observed reductions in metabolic activity. Although the PERMANOVA analysis did not detect significant global differences among all treatments (*p* = 0.505), a pairwise comparison revealed a significant difference between the carbendazim and control group (observed *p*-value = 0.030; permuted *p*-value = 0.032), supporting a treatment-specific proteomic response.

### 2.5. MALDI TOF/TOF MS Data Analysis

**Figure 7:**
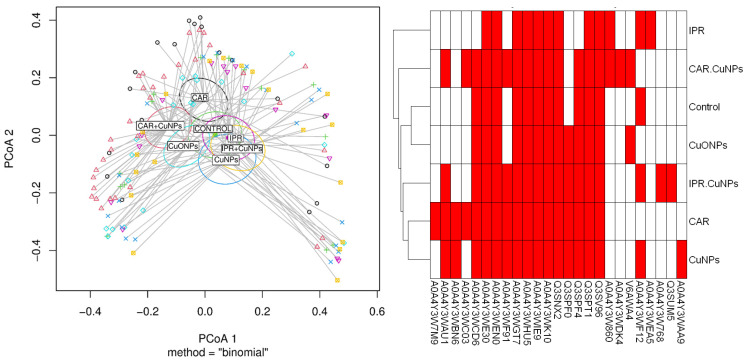
(**Left**) Cluster analysis (PCoA) of the PERMANOVA test performed for the main intensity values obtained from the MALDI-TOF MS/MS. Binomial method. (**Right**) Expression heatmap of the predicted proteins obtained from the UNIPROT database for *N*. *winogradskyi*.

The heatmap represents the predicted proteins found for each treatment (Figure 7, right), and the dendrogram represents the similarities between the treatments with the same entries. The molecular weights of the analytes were queried in the database, revealing peptide sequences with lengths ranging from 40 to 65 amino acid residues. Nonetheless, the N. winogradskyi database on UNIPROT showed that most proteins were not characterized. The cluster analysis is represented by a dendrogram (based on the Euclidean distance) on the left side of the heatmap. It suggested that the control and CuONPs had the most similar expression patterns, while the patterns for the iprodione and carbendazim treatments were more dissimilar than those of the control.

## 3. Discussion

*N. winogradskyi* plays a pivotal role in aerobic nitrification and nitrogen mobility, which are essential for maintaining environmental quality in agricultural soils. However, nitrification is a sensitive and slow process, and it is vulnerable to the introduction of anthropogenic chemical pollutants, which can potentially harm agricultural soil health [19]. Our findings indicated that the metabolic activity of *N. winogradskyi* was inhibited in the presence of the CuNPs and CuSO_4_ across all the tested concentrations. Conversely, the CuONPs demonstrated lesser inhibitory effects. This difference may be attributed to the distinct dissolution behaviors of the CuNPs and CuONPs in media. The release of ionic species, namely Cu^2+^ and Cu^+^, from nanoparticles is influenced by their oxidation into CuO(s) and is pH dependent [20]. Our results suggested that the lower solubility of the CuONPs in the nitrifying media (pH ≥ 7) resulted in the release of fewer copper ions compared to the CuNPs [21]. The distinct biological effects of Cu^+^ and Cu^2+^ ions stem from their transport mechanisms: Cu^+^ ions penetrate cells through passive diffusion, while Cu^2+^ ions predominantly remain in the extracellular space and bacterial periplasm [22]. The increase in Cu^+^ ions in the external media enhances their diffusion into bacterial cytoplasm, leading to oxidative stress through the formation of hydrogen peroxide and free radicals. This stress triggers the formation of DNA-oxo-copper complexes, causing DNA strand breaks and disrupting the transcription and replication processes [23]. Our data revealed a significant reduction in metabolic activity (approximately 95%) in *N. winogradskyi* when exposed to the CuNPs and CuSO_4_, whereas exposure to the CuONPs resulted in a minor reduction (approximately 69%). This observation implied that the copper ion’s oxidation state in the media was a crucial factor affecting *N. winogradskyi* metabolism.

Our findings also indicated that the CuNPs and CuSO_4_ harmed the metabolic activity of *N. winogradskyi* at concentrations below 2.5 mg L^−1^ for the CuNPs and 0.04 mM for the CuSO_4_. Supporting this, ref. [15] noted that nitrification kinetics could be affected at concentrations of 10 mg CuNPs L^−1^ or higher. Similarly, other studies have suggested that 5 mg of Cu L^−1^ (0.07 mM) can reduce the oxidizing activity of nitrobacter and nitrosomonas [24], aligning with our observed concentration range. The estimated IC_50_ value for the CuONPs (31.81 mg L^−1^) in our study on *N. winogradskyi* was significantly higher than that for the CuNPs (≤2.5 mg L^−1^), underscoring the importance of the copper nanoparticles’ oxidation states in dictating their toxicological impacts on *N. winogradskyi* metabolic activity.

In comparison, copper concentrations vary widely, ranging from 5 to 30 mg Cu kg^−1^ in grasslands, forests, and agricultural soils to 100 to 300 mg Cu kg^−1^ in vineyards and orchards [25]. However, it is essential to note that the biologically available exchangeable copper ions may differ from the total soil content. In contrast to copper compounds, our results indicated that the pesticides iprodione, 2,4-D, and carbendazim were less toxic. Ref. [26] reported that carbendazim might be safe for nitrification at concentrations as high as 220 mg kg^−1^ (1.15 mM), significantly above the recommended field rate. However, ref. [27] suggested that concentrations as low as 50 mg kg^−1^ (0.29 mM) could negatively impact soil nitrifiers. Iprodione has been reported to alter soil bacterial communities, including nitrifiers [28], while 2,4-D, according to [29], appears not to be toxic to bacterial metabolism. However, its effects on nitrobacter are not documented.

The toxicity of the pesticide derivatives, including catechol, 3,5-dichloroaniline, and 2,4-dichlorophenol, was higher than that of the parent pesticides on *N. winogradskyi*. For instance, 3,5-dichloroaniline was more toxic than iprodione, with an IC50 value of 0.26 mM, and it is known to be harmful to soil microorganisms [30]. Klein and Tenno [31] estimated its IC_50_ value for nitrification inhibition at 2.9 mg L^−1^ (0.0179 mM). Catechol, derived from carbendazim, also inhibited metabolic activity, with an IC50 value of 0.74 mM, which contrasted with the lower value of 0.09 mM suggested by [32]. Similarly, 2,4-dichlorophenol (derived from 2,4-D) exhibited an IC50 value of 0.85 mM against *N. winogradskyi,* while [33] reported a lower IC50 value of 0.22 mM for *Nitrobacter* sp.

The inhibitory effects on metabolic activity were also demonstrated in the mixtures of pesticides and copper nanoparticles. All mixtures, except carbendazim + CuONPs and iprodione + CuONPs, had estimated IC_50_ values of ≤0.04 mM. The Loewe additivity model suggested that the interactions were mainly additive, with an antagonistic effect observed for the mixture of 0.04 mM iprodione + 2.5 mg L^−1^ CuNPs. The RSH analysis identified the CuNPs, CuONPs, and CuSO_4_ as the primary factors impacting metabolic activity in the iprodione mixtures rather than iprodione. These observations underscored the significant impact of copper on metabolic activity.

It is important to note that the combined treatments were tested at fixed 1:1 molar ratios. While this design allowed us to assess the interaction effects at a specific and controlled concentration point, it did not capture the possible shifts in interaction type (e.g., from synergistic to antagonistic) that may have occurred across the different concentration ratios. Future studies incorporating a broader matrix of mixture ratios would be valuable for better understanding the dynamic nature of these interactions.

The scarcity of studies on the impacts of co-contamination on nitrifying organisms makes comparisons challenging [34]. Our findings highlight the sensitivity of *N. winogradskyi* to copper nanoparticles, which may impact ammonia retention and contribute to eutrophication in agricultural soils. The data also suggested that pesticide derivatives and their mixtures with CuNPs could harm *N. winogradskyi* metabolic activity. It is worth noting that pairwise Student’s *t*-tests were used to assess specific concentration-dependent effects. Although more integrative approaches such as ANOVA followed by a post hoc analysis could offer broader statistical insights, the applied tests were complemented by IC_50_ estimations and interaction analyses (e.g., Scheirer–Ray–Hare), which collectively supported the robustness of our conclusions within the scope of our study. This statistical approach was consistent with the methodologies previously applied in microbial inhibition assays using WST-1-based metabolic indicators in microplate formats, as demonstrated by Johnsen et al. [35]. Still, more research is needed to understand the total bioavailable copper from nanoparticles and its interaction with soil constituents, such as humic acid and microbial activity.

The statistical analysis of the protein profiles using MALDI-TOF/MS revealed minimal variations, except for carbendazim, compared to the control. However, the presence–absence protein matrix revealed a joint protein group across all treatments. The most similar protein-expression profiles were observed in the control and CuONP treatments, indicating minimal differences in cellular response. In contrast, the control versus CuNPs and control versus carbendazim treatments showed more significant differences, indicating varied cellular reactions. The comparison between iprodione and carbendazim highlighted the differences in protein presence or absence, suggesting divergent cellular responses.

While the proteins identified remain uncharacterized (as detailed I the Supplementary Materials, Table S1), this limitation paralleled the challenges of functional annotation in the proteome analysis of *N. winogradskyi*. Similar approaches have been used in studies on *Klebsiella pneumoniae* exposed to antibiotics [36] and on *Escherichia coli* under heat stress [37] but not for assessing the effects of pesticides on *N. winogradskyi*.

The results obtained in this study suggest that *N. winogradskyi* activity can be significantly suppressed in the field by similar concentrations of CuNPs as those of pesticides. However, the challenge lies in determining the total concentration of bioavailable Cu^2+^ from the CuNPs in soil, considering their distinct chemical behavior compared to culture media [38].

Given the widespread use of copper as an active ingredient in pesticides for controlling fungal diseases and delivering micronutrients to crops, its accumulation in soil over decades poses significant concerns. As suggested by other researchers, the beneficial opportunity of using CuPs in agriculture may be limited, as low levels can disrupt nitrification kinetics. In contrast, excessive use can harm plant nutrition [15]. The inappropriate application of CuNPs could cause collateral damage to crops and soil microorganisms, considering the presence of similar nitrifying organisms in the soil. This study contributes valuable information for environmental risk assessment policies regarding the agricultural use of CuNPs in conjunction with pesticides, particularly concerning their impact on nitrifying organisms.

The interaction between copper-based nanoparticles and pesticides revealed distinct toxicity profiles depending on the combination. Individually, the CuNPs and CuSO_4_ showed potent inhibitory effects on *N. winogradskyi*, while the CuONPs and the tested pesticides displayed slight or negligible toxicity. However, when combined, several mixtures demonstrated enhanced toxicity, particularly the CuNPs with carbendazim, iprodione, or pesticide derivatives, often exceeding the effect of the CuNPs alone. This suggests that CuNPs act as the dominant toxic component, possibly facilitating the cellular uptake or bioavailability of co-applied pesticides. The IC_50_ values of the combined treatments were frequently lower than those of the individual pesticides, and the Loewe additivity model confirmed mostly additive effects, with isolated cases of antagonism or synergism depending on the compound. These patterns pointed to a primarily non-interfering additive toxicity mechanism, although the physicochemical interactions at the nanoparticle–pesticide interface may have modulated specific responses. Understanding these dynamics is crucial for predicting environmental risks in agricultural systems where such co-exposures are increasingly common.

Previous studies on nitrification under exposure to metallic nanoparticles support the findings observed here for *N. winogradskyi*. For example, soil microcosm assays have shown that CuNPs can significantly reduce nitrification rates, likely by affecting the activity of nitrifying bacteria as a whole [15]. Additionally, *Nitrospira* spp., often the dominant NOBs in soil systems, have exhibited sensitivity to metal pollutants and pesticides, though with slightly higher resilience compared to *Nitrobacter* [14]. These observations align with our results and suggest that NOBs, mainly *Nitrobacter,* may serve as early bioindicators of chemical stress in contaminated environments. The consistent inhibition across the different nitrifying groups reinforced the vulnerability of the nitrite oxidation step in the nitrogen cycle under nanoparticle and pesticide exposure.

Although this study was performed using a pure culture system, the marked inhibition of this strain by the CuNPs and their mixtures with pesticides suggests a potential disruption in the nitrification process in soils. Specifically, the impaired conversion of nitrite to nitrate may result in nitrite accumulation, which can be toxic to plants and alter microbial community dynamics. This imbalance could cascade through the nitrogen cycle, affecting ammonia oxidation by feedback inhibition and disrupting nitrate availability for plant uptake and the denitrification processes. In natural soil ecosystems, where nitrifying bacteria operate within complex microbial networks, such inhibition may contribute to shifts in community composition, reduced nitrogen-use efficiency, and an increased risk of nitrogen loss or the accumulation of reactive nitrogen species. Therefore, these findings highlight the importance of evaluating pollutant impacts on key functional microbial groups within the broader context of soil nitrogen cycling.

The combined effects observed in this study are consistent with reports on co-contamination scenarios, including metallic nanoparticles and pesticides in soil. Tortella et al. [16] demonstrated that CuNPs can significantly alter the sorption and dissipation patterns of carbendazim and iprodione in agricultural soils, increasing their persistence and modifying microbial activity. Similarly, Parada et al. [17,18] reported additive or synergistic toxicity effects when CuNPs and atrazine were applied together, disrupting the abundance of nitrifying microorganisms and affecting soil nitrogen transformations. These findings parallel the additive effects seen in our study and highlight the potential for CuNPs to act as facilitators of pesticide bioavailability or as co-stressors in microbial systems. Such interactions are particularly relevant in intensive agricultural systems, where the co-occurrence of nanomaterials and agrochemicals is increasingly frequent.

Finally, it is acknowledged that a time-series analysis could yield a more comprehensive understanding of the progression and potential adaptation of *N. winogradskyi* to these pollutants. Future studies incorporating multiple time-points would help elucidate the temporal dynamics of microbial response and resilience under combined chemical stress.

## 4. Materials and Methods

### 4.1. Chemical Compounds

The chemical compounds used in this work were CuNPs (40–60 nm, SkySpring Nanomaterials Inc., Houston, TX, USA), CuONPs (40–60 nm, SkySpring Nanomaterials Inc.), and copper(II) sulfate (CuSO_4_·7H_2_O). The CuNPs used were previously characterized [39].

Prior to the experiments, the CuNPs and CuONPs were characterized to determine their physicochemical properties in aqueous suspensions. A dynamic light scattering (DLS) analysis revealed hydrodynamic sizes (Z-average) of 70.08 nm for the CuNPs and 77.02 nm for the CuONPs, with polydispersity index (PdI) values of 0.277 and 0.283, respectively. The measured zeta potentials were +17.7 mV for the CuNPs and +30.5 mV for the CuONPs, indicating moderate colloidal stability. These measurements were performed in molecular-grade water under the same conditions used for the nanoparticle dispersion before the biological assays. Detailed size distribution profiles are provided in Supplementary Figure S1.

The analytical pesticide standards, including iprodione, carbendazim, and 2,4-D (99.9% Pestanal^®^), were purchased from Sigma-Aldrich (Merck). The pesticide derivatives—3,5-dichloroaniline (pestanal^®^), catechol (phyproof^®^), and 2,4-dichlorophenol (99%, Sigma-Aldrich)—were purchased from Sigma-Aldrich (Merck). All chemical compounds were purchased from Merck unless otherwise stated.

### 4.2. Culture Conditions

The *Nitrobacter winogradskyi* DSM-10237 was obtained from the Leibniz Institute DSMZ–German Collection of Microorganisms and Cell Cultures GmbH (Germany). Axenic cultures of *Nitrobacter winogradskyi* were cultivated at an initial cell density of 1 × 10^6^ cells mL^−1^ for 30 days at 130 rpm and 30 °C in the dark using an autotrophic medium specific for *Nitrobacter* (German Collection of Microorganisms and Cell Cultures, DSMZ medium 756c). The medium per liter contained the following: 2 g NaNO_2_, 100 mL of stock solution (composed of 5 g NaCl, 1.5 g KH_2_PO_4_, 0.5 g MgSO_4_·7H_2_O, and 0.07 g CaCO_3_ per liter), and 1 mL of trace element solution (per liter: 97.3 mg FeSO_4_·7H_2_O, 49.4 mg H_3_BO_3_, 43.1 mg ZnSO_4_·7H_2_O, 37.1 mg (NH_4_)_6_Mo_7_O_24_·4H_2_O, 33.8 mg MnSO_4_·2H_2_O, and 25.0 mg CuSO_4_·5H_2_O). The pH was adjusted to 7.5. Then, 2 mL of culture was collected every 48 h to assess the bacterial growth. One milliliter was used to measure the optical density at 600 nm (OD_600_) using a UV-Vis Spectrophotometer (Bausch & Lomb, Model TU-1810 Split Beam, Rochester, NY, USA). The remaining 1 mL was diluted in 9 mL of sterile water and stained with 10 μL of acridine orange (125 mg mL^−1^). After 5 min of incubation, the sample was filtered through a black polycarbonate membrane filter (pore size: 0.22 μm) for bacterial cell counting by epifluorescence microscopy, following the method in [40].

### 4.3. Metabolic Activity Assay

A 500 mL culture media of *N. winogradskyi* containing 1 × 10^7^ bacteria mL^−1^ was centrifuged at 10,000× *g* for 10 min. Aliquots of 115 μL of an *N. winogradskyi* suspension (1 × 10^7^ bacterium mL^−1^) were transferred into 96-well microplates (Ultra Cruz) and supplemented with the CuNPs (2.5, 10.2, 40.7, or 162.7 mg L^−1^), CuONPs (3.2, 12.7, 50.9, or 203.6 mg L^−1^), or CuSO_4_ (0.04, 0.16, 0.64, or 2.56 mM). All assays were done in triplicate and used field concentrations, as suggested in [41]. Additionally, the *N. winogradskyi* was exposed to incremental concentrations of the pesticides (0.04, 0.16, 0.64, or 2.56 mM), as suggested in [26,41,42,43]. The pesticide derivatives also used incremental concentrations (0.04, 0.16, 0.64, or 2.56 mM). The combination of nanoparticles, pesticides, or pesticide derivatives followed the exact arrangement of informed concentrations at a 1:1 ratio. All the individual treatments with the copper-based compounds (CuNPs, CuONPs, and CuSO_4_) and pesticides (iprodione, carbendazim, and 2,4-D) were tested independently. These served as controls and baseline references to interpret the mixture effects, and their responses were used for IC_50_ determination and interaction analysis using Loewe additivity and Scheirer–Ray–Hare models.

After 8 days of incubation at 30 °C in a humid chamber, 10 μL of tetrazolium salt (WST-1) was added to each well and incubated for 12 h. Optical density (OD) at 480 nm was measured to assess the reduction of WST-1 to formazan (Amax 420–480 nm) [35]. The OD values were normalized using the control as 100%. The results were expressed using the following nomenclature: treatments with unique compounds (treatments containing only one assayed compound, such as a pesticide derivative or nanoparticles), balanced treatments (treatments with the same concentration for both compounds), and unbalanced treatments (treatments with two compounds at different concentrations).

### 4.4. Protein Profile of N. winogradskyi Exposed to CuNPs and Pesticides

The protein profile of *N. winogradskyi* was analyzed using matrix-assisted laser desorption/ionization (MALDI) time-of-flight (TOF)/TOF mass spectrometry (MS). For this test, 2 L of N. winogradskyi with a concentration of approximately 1.0 × 10^8^ bacteria per mL^−1^ in PBS buffer was used. A volume of 285 µL of *N. winogradskyi* was mixed with 715 µL of nitrifying medium and a concentration of the pesticide compounds corresponding to IC_50_ for the CuNPs, CuONPs, carbendazim, iprodione, carbendazim + CuNPs, and iprodione + CuNPs was used to obtain 1000 µL of the total volume. The assay included a control and 6 treatments. All assays were incubated for 24 h at 30 °C. Then, the samples were rinsed with molecular-grade water (ultrapure water free of nucleases and organic contaminants) and centrifuged at 8000× *g* for 2 min. The supernatant was discarded, and the cellular pellets were used. The bacterium cells were resuspended in 20 µL of formic acid, and 20 µL of acetonitrile was added. The samples were centrifuged at 8000× *g* for 2 min. Aliquots of 1 µL of supernatant from each sample were transferred onto a MALDI plate and dried at room temperature, then 1 µL of a saturated solution of alpha-cyano-4-hydroxy-cinnamic acid (diluted in 50% acetonitrile, 47% water, and 2.5% trifluoroacetic acid) was added on the surface of the plate, which contained the sample.

The mass spectrum of the protein samples was obtained using MALDI-TOF/TOF MS Autoflex Speed (Bruker Daltonics, Bremen, Germany) with a brilliant beam laser (334 nm). All analyses were performed using linear mode with positive polarity, a voltage acceleration of 20 kV, and an extraction delay of 220 ns. Each spectrum was collected as the mean of 1200 laser beam shots, with sufficient energy to achieve spectrum resolution without saturation, in the 2000 to 20,000 mass-to-charge ratio range. The instrument was externally calibrated using a protein standard (Protein Calibration Pattern I-Bruker Daltonics, Bremen, Germany), which comprised insulin, ubiquitin, cytochrome C, and myoglobin.

The mass-to-charge ratios of the proteins obtained by a MALDI-TOF/TOF MS analysis were compared using UniProtKB/Swiss-Prot with the TagIdent Tool (https://web.expasy.org/tagident (accessed on 23 October 2024). A list from the database was acquired, and the search criteria employed were “protein molecular weight: 1000–10,000 Da, taxonomy: *Nitrobacter winogradskyi*.” [44].

### 4.5. Statistical Analysis

The acquired data were processed using MS Excel and RStudio (R version 4.0.1). A one-way analysis of variance (ANOVA), followed by a Tukey’s post hoc test, was applied to assess the significant differences (*p* < 0.05) among the concentrations within each treatment group. Additionally, paired Student’s *t*-tests were conducted for the selected pairwise comparisons of biological relevance. Linear regressions were applied to the normalized activity levels using the R programming language in RStudio to determine the IC_50_ for each treatment. The Scheirer–Ray–Hare test (SRH) [45] was utilized to evaluate the effects of the factors on the compound combinations, with significance set at *p* < 0.05. The unbalanced treatments, theoretically derived, were calculated from the modeled values using non-linear regression (a negative binomial distribution). These data were then incorporated into a biological response matrix. The synergistic effects were assessed using Loewe additivity [46], which involved generating isobolograms and additivity tables. The normalized responses in the modeled isobolograms ranged from 0 (indicating low activity) to 1 (denoting the highest activity) relative to the control. The MALDI-TOF/TOF MS spectra were analyzed using a permutational multivariate analysis of variance (PERMANOVA), with 999 permutations for the F-statistic and a significance threshold of *p* < 0.05. The prominent signal peaks (>500 *m*/*z*) were characterized by four parameters: mass-to-charge ratio, signal intensity, quality factor, and signal-to-noise ratio. A distance matrix encompassing both the binomial and Euclidean distances served as the foundation for the clustering analyses. The graphical representation of these analyses was executed using principal coordinates analysis (PCoA) [47].

## 5. Conclusions

The results obtained in this study demonstrate that both CuNPs and CuONPs can negatively impact the metabolic activity of *N. winogradskyi*, a key nitrite-oxidizing microorganism in the nitrogen cycle. The CuNPs exhibited significantly higher toxicity than the CuONPs, pesticides, or their derivatives. Moreover, combinations of the CuNPs with pesticides or degradation products frequently resulted in additive or even enhanced toxicity, as shown by the Loewe additivity analysis. These findings suggest that CuNPs may pose a considerable risk to nitrifying microorganisms, potentially disrupting soil nitrogen dynamics when applied at non-targeted or excessive levels. Given the projected increase in nanoparticle use in agriculture, future research should focus on defining environmentally safe concentrations, exploring time-dependent effects, and evaluating impacts in complex soil microbiomes under realistic field conditions.

## Figures and Tables

**Table 1:** Calculated IC_50_ (50% inhibition of metabolic activity) values for the copper-based nanoparticles, pesticides, and degradative products.

Evaluated Compounds	Mixed Treatment Type	Estimated IC_50_ *
CuNPs	Copper-based comp.	≤2.5 mg L^−1^
CuONPs	“	31.81 mg L^−1^
CuSO_4_	“	≤0.04 mM
Carbendazim	Pesticide (fungicide)	≥2.56 mM
Iprodione	“	0.83 mM
2,4-D	Pesticide (herbicide)	≥2.56 mM
3,5-dichloroaniline	Pesticide derivate	0.26 mM
Catechol	“	0.74 mM
2,4-dichlorophenol	“	0.85 mM
Carbendazim + CuNPs (1:1)	Fungicide + copper-based comp.	≤0.04 mM + ≤2.5 mg L^−1^
Carbendazim + CuONPs (1:1)	“	0.92 mM + 73.18 mg L^−1^
Carbendazim + CuSO_4_ (1:1)	“	≤0.04 mM
Iprodione + CuNPs (1:1)	“	≤0.04 mM + ≤2.5 mg L^−1^
Iprodione + CuONPs (1:1)	“	1.26 mM + 100.22 mg L^−1^
Iprodione + CuSO_4_ (1:1)	“	≤0.04 mM
2,4-D + CuNPs (1:1)	Herbicide + copper-based comp.	≤0.04 mM + ≤2.5 mg L^−1^
3,5-dichloroaniline + CuNPs (1:1)	Pesticide derivate + copper-based comp.	0.08 mM + 5.08 mg L^−1^
Catechol + CuNPs (1:1)	“	0.46 mM + 29.23 mg L^−1^
2,4-dichlorophenol + CuNPs (1:1)	“	≤0.04 mM + ≤2.5 mg L^−1^

* The nanoparticle concentrations are expressed in mg L^−1^, while the pesticide and derivative concentrations are expressed in mM. This reflects the different physicochemical natures of the substances. The nanoparticle concentrations could not be converted to molarity as they did not consist of discrete molecular species.

## Data Availability

Data are contained within the article and Supplementary Materials.

## Supplementary Materials

The following supporting information can be downloaded at: https://www.mdpi.com/article/10.3390/ijms26136391/s1.
