# Supplementary material for: Impact of Combined Exposure to Copper Nanoparticles, Copper Oxide Nanoparticles, and Pesticides on the Metabolic Activity of Nitrobacter winogradskyi
Source: Int J Mol Sci. 2025 Jul 2;26(13):6391. doi: 10.3390/ijms26136391 (PMC12249886; doi:10.3390/ijms26136391)
Supplement: Supplementary file 1 [file ijms-26-06391-s001.zip › ijms-3714703-supplementary.pdf]

Supplementary Materials

Table S1. List of queried proteins at UNIPROT database.

UNIPROT ID	Status	Gene	Protein name	Length	Mass	pI
A0A4Y3WAA9	unrev.	NWI01_14270	Uncharacterized protein	47	5649.468	9.307171
Q3SUM5	unrev.	Nwi_0751	HIG1 domain-containing protein	64	7083.823	12.80743
A0A4Y3W768	unrev.	NWI01_03400	Uncharacterized protein	62	6917.041	9.866122
A0A4Y3WEA5	unrev.	NWI01_28770	Uncharacterized protein	51	5675.564	10.56615
A0A4Y3WF12	unrev.	NWI01_33750	Uncharacterized protein	63	6977.117	12.14132
V6AWA4	unrev.	Tag enc. tmRNA	Proteolysis tag peptide encoded tmRNA (Frag.)	12	1204.246	3.749972
A0A4Y3WDK4	unrev.	NWI01_30040	Uncharacterized protein	62	6976.812	11.1857
A0A4Y3W860	unrev.	NWI01_10760	Uncharacterized protein	65	7024.172	5.723676
Q3SV96	unrev.	Nwi_0528	Uncharacterized protein	66	6934.013	10.31935
Q3SPT1	unrev.	Nwi_2457	Uncharacterized protein	50	5538.275	4.66497
Q3SPF4	unrev.	Nwi_2584	Uncharacterized protein	51	5547.944	10.45513
Q3SPF0	unrev.	Nwi_2588	Uncharacterized protein	62	7017.03	11.53345
Q3SNX2	unrev.	Nwi_2766	Uncharacterized protein	51	5626.935	11.4308
A0A4Y3WK10	unrev.	NWI01_35680	Uncharacterized protein	52	5582.612	8.239544
A0A4Y3WIE9	unrev.	NWI01_35610	Uncharacterized protein	42	4434.188	6.425915
A0A4Y3WHU5	unrev.	NWI01_33710	Uncharacterized protein	52	5524.358	5.669753
A0A4Y3WGT7	unrev.	NWI01_32120	Uncharacterized protein	51	5556.259	10.47183
A0A4Y3WF91	unrev.	NWI01_27130	Uncharacterized protein	50	5482.17	9.165677
A0A4Y3WEN0	unrev.	NWI01_15880	Uncharacterized protein	63	6955.315	6.647179
A0A4Y3WE30	unrev.	NWI01_25840	Uncharacterized protein	51	5592.919	11.4308
A0A4Y3WCD6	unrev.	NWI01_25840	Uncharacterized protein	50	5491.285	9.347669
A0A4Y3WC03	unrev.	NWI01_23460	Cytochrome c oxidase subunit IV	50	5471.194	4.225549
A0A4Y3WBN6	unrev.	NWI01_17700	Uncharacterized protein	61	6971.841	4.828015
A0A4Y3WAU1	unrev.	NWI01_20490	Uncharacterized protein	51	5633.245	4.401677
A0A4Y3W7M9	unrev.	NWI01_08860	Uncharacterized protein	47	5649.468	9.307171

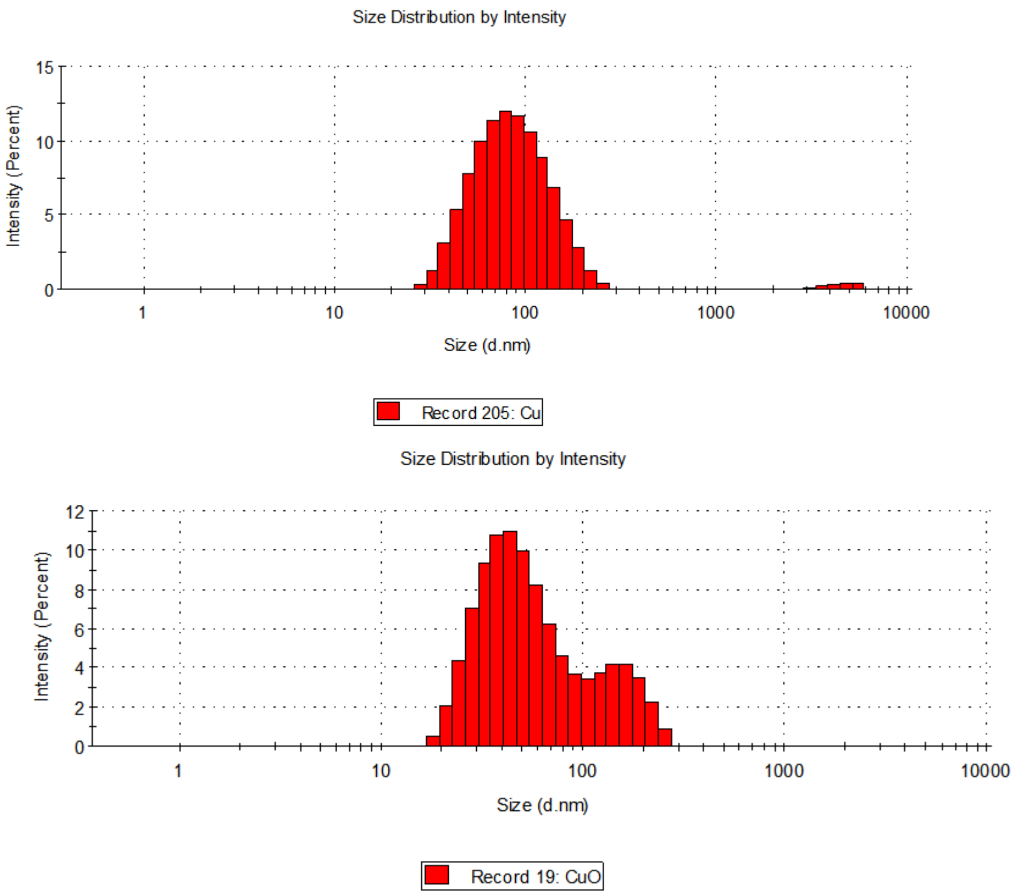

Nanoparticles (40–60 nm)	DLS size [Z-average (d.nm)]	(PdI)	(mV)
**Cu**	70.08	0.277	+ 17.7
**CuO**	77.02	0,283	+30.5

Figure S1. Size distribution of CuO NPs and Cu NPs and in aqueous media, Polydispersity index (PdI) and Zeta potential (mV).
